# Brahma-Related Gene 1 Deficiency in Endothelial Cells Ameliorates Vascular Inflammatory Responses in Mice

**DOI:** 10.3389/fcell.2020.578790

**Published:** 2020-11-30

**Authors:** Yuanyuan Zhang, Huidi Wang, Mingzi Song, Tongchang Xu, Xuyang Chen, Tianfa Li, Teng Wu

**Affiliations:** ^1^Key Laboratory of Targeted Intervention of Cardiovascular Disease, Department of Pathophysiology, Collaborative Innovation Center for Cardiovascular Translational Medicine, Nanjing Medical University, Nanjing, China; ^2^Department of Cardiology, The First Affiliated Hospital of Hainan Medical University, Haikou, China; ^3^Laboratory Center for Experimental Medicine, Jiangsu Health Vocational College, Nanjing, China

**Keywords:** BRG1, endothelium, diabetic atherosclerosis, abdominal aortic aneurysms, inflammation

## Abstract

Endothelial dysfunction plays an important role in promoting the progression of disease genesis such as atherosclerosis and abdominal aortic aneurysm (AAA). The physiological unbalance of endothelial cells is a major pathological basis. In this present study, we investigated Brahma-related gene 1 (BRG1), a chromatin remodeling protein, was in mouse models of diabetic atherosclerosis and AAA, focusing on its role in endothelial dysfunction. We report that compared with their wild-type (WT, ApoE*^–/–^*; BRG1*^*fl/fl*^*) littermates, endothelium conditional BRG1 knockout mice (CKO, ApoE*^–/–^*; BRG1*^*fl/fl*^*; CDH5*-cre*) exhibited an alleviated phenotype of diabetic atherosclerosis. Immunohistochemically staining and real-time PCR analysis demonstrated fewer macrophages recruitment with a reduction of vascular inflammatory in CKO mice compared with WT mice. Further research in the Ang-II induced AAA model revealed that BRG1 deficiency had the protective effects on endothelium conditional BRG1 deletion, evidenced by the downregulation of pro-inflammatory mediators [interleukin (IL)-1β and IL-6, not tumor necrosis factor-α (TNF-α)] in the vessels of CKO mice compared with WT mice. In Ea.hy926 cell lines, anti-BRG1 small interfering RNA and PFI-3 treatment obviously alleviated tumor necrosis factor-α-induced IL-6 and CCL2 expression, and further research demonstrated that the BRG1 inhibition in endothelial cells not only decreased c-Fos expression but also blocked the c-Fos translocation into nuclei. In conclusion, our results suggest that endothelial BRG1 deficiency may protect the mice from diabetic atherosclerosis and AAA via inhibiting inflammatory response in vessels.

## Introduction

Endothelium, a continuous monolayer of endothelial cells, separates the circulation and the vascular basal lamina under physiological conditions (Kruger-Genge et al., 2019). Endothelial injury, also called endothelial dysfunction, is a prominent feature in many cases and directly causes vascular injury. Endothelial injury is key to the initiation and progression of severe vascular diseases, including atherosclerosis and aneurysms (Czubryt, 2015). In these diseases, injured endothelial cells mediate the inflammatory response, which further inducing recruitment of macrophages, proliferation and migration of smooth muscle cells, and expressing adhesion molecules, finally promoting the progression of the disease (Li H. et al., 2018; Maguire et al., 2019).

Diabetes mellitus affects more than 180 million people worldwide. Diabetic patients exhibit significantly higher risk for cardiovascular disease (CVD) (Boyle et al., 2001). Diabetes is often accompanied by synergistic risk factors such as hypertension, obesity, systemic inflammation, hypercoagulability, and dyslipidemia, which further increase CVD death rates (Stamler et al., 1993). For instance, there is a growth of evidence that also showed a higher prevalence of arteriosclerosis in people with diabetes. In addition, numerous observational studies have found increased levels of the mediators of inflammation, such as C-reactive protein, interleukin-6 (IL-6), and plasminogen activator inhibitor 1, to name only a few, as major associative findings between diabetes and atherosclerosis (La Sala et al., 2019). Insulin resistance, a prominent feature of type 2 diabetes mellitus, has been demonstrated as an important risk factor for inducing atherosclerosis among the patients diagnosed with diabetes even in the absence of hyperglycemia (DeFronzo, 2010). Interestingly, previous research also found the insulin receptors in vascular endothelial cells, which indicates the role of insulin in the regulation of vascular endothelial cells (Han et al., 2011; Pansuria et al., 2012). Further studies also proved that hyperglycemia induced excessive endothelial inflammatory response. It has been considered as the main reason for the endothelial injury, which further promotes diabetes mellitus-associated CVDs (Fadini et al., 2016). For instance, recent research proposed that inflammatory response induced by Nod-like receptor family pyrin domain containing 3 inflammasome or excessive reactive oxygen species (ROS) levels could induce endothelial injuries to promote atherosclerosis (Wang R. et al., 2017; Wan et al., 2019), a chronic inflammatory disease that can give rise to various CVDs. In this case, inflammatory cells and activated endothelial cells in atherosclerotic plaques upregulate adhesion molecules and release inflammatory cytokines such as tumor necrosis factor-α (TNF-α), IL-6, and IL-1β, promoting the progression of atherosclerosis (Siti et al., 2015). Statin therapy, traditional clinical therapeutic strategies for diabetes related atherosclerosis, was statistically significantly associated with reductions in the incidence of atherosclerotic CVD-caused mortality in the presence of diabetes. This effect decreased after age 85 years and disappeared in nonagenarians (Ramos et al., 2018). However, as the most frequently used drugs for atherosclerosis treatment, statin therapy may cause such adverse effects such as myopathy (Collins et al., 2016). Recently, preclinical research that suggested anti-inflammatory strategies such as metformin, berberine, or raising apolipoprotein AI levels were effective in atherosclerosis treatment for animal models (Barrett et al., 2019; Tang et al., 2019), which proved the effect of diabetic atherosclerosis treatment for anti-inflammatory strategy.

Pathologically, the features of abdominal aortic aneurysm (AAA) are complicated, including transmural inflammatory infiltration, noticeable breakdown of elastic lamellae, smooth muscle cell loss, and endothelial cell death and detachment (Moxon et al., 2010; Qin et al., 2013). The pathological changes of endothelial cells or endothelial dysfunction are possibly earlier than those of media and adventitia in the process of AAA formation. Heterogeneous research has shown that endothelial dysfunction promoted AAA pathogenesis via different factors, among which aberrant inflammation plays a key role (Dale et al., 2015). For example, ample evidence supports endothelial dysfunction, which enhanced the recruitment of circulating macrophages, finally leads to augment production and release of matrix metalloproteinases (MMPs), which in turn degrade elastin and disintegrate the medial layer (Samadzadeh et al., 2014). Inflammatory cytokines, such as IL-1β, IL-6, and TNF-α, have been reported to be significantly upregulated in patients with AAA (Lamblin et al., 2010; Golledge, 2019). These inflammatory mediators stimulate the expression of MMP-2 and MMP-9 in vessels, which lead to the damage of vascular wall integrity through inducing extracellular matrix degradation (Samadzadeh et al., 2014; Mallat, 2017). Anti-inflammatory treatment such as inhibition of the mammalian target of rapamycin pathway or genetic ablation of microRNA-33 attenuates inflammation and AAA (Li et al., 2017; Nakao et al., 2017), indicating that anti-inflammatory treatment should be the promising strategy for AAA treatment.

In the present investigation, we sought to determine the role of endothelial Brahma-related gene I (BRG1) in vascular inflammation in animal models of diabetes-related atherosclerosis or AAA. BRG1 is the catalytic subunit of the SWI/SNF chromatin remodeling complex and regulates gene expression via adenosine triphosphate hydrolysis-driven chromatin remodeling (Xu and Fang, 2012). Recent studies have found that BRG1 regulates heart muscle development in mice (Xiao et al., 2016). BRG1 can also regulate myocardial ischemia–reperfusion injury via inhibiting the inflammatory response and ROS production (Li Z. et al., 2018). Previous research demonstrated that endothelial-specific BRG1 knockout in mice could ameliorate the progression of atherosclerosis and AAA formation (Fang et al., 2013; Zhang et al., 2018a). However, the mechanism of BRG1 in regulating diabetes-related macrovascular cardiovascular atherosclerosis and Ang II induced AAA formation under *ApoE* gene knockout condition remain not to be proved. Here, we report that BRG1 deletion in endothelial cells blocked the progression of diabetes mellitus-related atherosclerosis and Ang II-induced AAA model under the *ApoE* gene knockout condition via inhibiting c-Fos expression as well as blocking c-Fos nucleic translocation, which further inhibits inflammatory response in endothelial cells. Our data hopefully may extend the current knowledge regarding the BRG1 function in CVDs.

## Materials and Methods

### Ethics

The studies involving animals were reviewed and approved by the intramural Committee on Ethical Conduct of Animal Studies of Nanjing Medical University.

### Reagents

Oil-red O powder was purchased from Sigma Aldrich (St. Louis, MO, United States, O0625). All the real-time polymerase chain reaction (PCR) primers were purchased from Sangon (Shanghai, China). Hematoxylin–eosin (H&E) stain kit was purchased from Beyotime Biotechnology Co. (Beijing, China, C0105). RNA extraction kit and real-time PCR kit were purchased from Vazyme Biotech Co., Ltd. (Nanjing, China, Q311). Streptozotocin (STZ) was purchased from Selleck Co. (Shanghai, China, S1312).

### Animal Feeding

Homozygous apolipoprotein E-deficient mice (ApoE*^–^*^/^*^–^*) were obtained from the Jackson Laboratory. According to the latest report, BRG1*^*fl/fl*^*; CDH5*-cre* mice crossed by Brahma related gene 1-loxp (BRG1*^*fl/fl*^*) mice and CDH5*-cre* mice were obtained from Nanjing Biomedical Research Institute of Nanjing University. ApoE*^–/–^* and BRG1*fl/fl*; CDH5*-cre* mice were crossed to obtain ApoE*^–^*^/^*^–^*; BRG1*^*fl/fl*^*; CDH5-cre mice. The F1 progeny of this mating (ApoE^±^; BRG1*^*fl/*+^*; CDH5*^–*cre*^*) was crossed to obtain ApoE*^–/–^*; BRG1*^*fl/fl*^*; CDH5*-cre* [conditional knockout (CKO)] mice and their littermate control ApoE*^–^*^/^*^–^*; BRG1*^*fl/fl*^* [wild-type (WT)] mice. All offspring were genotyped by PCR techniques and lived in specific pathogen-free conditions in accordance with the guidelines from the National Institutes of Health Guide for the Care and Use of Laboratory Animals in China.

### Animal Model Construction

To induce diabetes-accelerated atherosclerosis, 8 weeks old male CKO and WT mice were intraperitoneally injected STZ for 5 days, then high-fat diets containing 40 kcal% fat, 1.25% cholesterol, 0.5% cholic acid (Research Diets, United States, D12109) bred for last 4 weeks. Ang II was utilized to induce mice AAA models; 8 weeks old male CKO and WT mice were randomly allocated to Ang II infusion or control, and mini-osmotic pumps (Alzet, Cupertino, CA) containing Ang II (1,000 ng/min per kg mice, Sigma, A9525) or saline were used by infusing Ang II or saline for 28 days using published protocols. Before killing, vessel diameter was determined by ultrasonography using a Vevo 660 imaging system (VisualSonics). Two-dimensional images (B mode) of the short-axis scan were acquired to determine the maximal diameters of suprarenal aortas. The abdominal aorta was immediately excised, photographed, and analyzed histologically.

### Survival Rate, Body Weight, and Postprandial Blood Glucose Detection

The data of survival rate were collected after feeding up with high-fat diet. Before collecting mice tissue samples, we collected the data of mice’s body weight, and postprandial blood glucose was measured directly from the tail tip with a glucometer.

### Isolate Vascular Ring Function Experiment

Mice were killed using an overdose of ethyl ether and perfused with phosphate-buffered saline (PBS). The thoracic/abdominal aortas were separated from fat or other tissues. Then, the aortas were put into DMT 620M. Vasorelaxation of isolated aortic ring segments was determined in the oxygenated Krebs’ solution. After an equilibration period of 60 min, aortic rings were stimulated to contract with contracted norepinephrine (10^–7^ M). Endothelium-dependent or independent relaxation was then assessed in response to a cumulation of acetylcholine (10^–9^–10^–5^ M) or sodium nitroprusside (SNP, 10^–9^–10^–5^ M). Relaxation at each concentration was measured and expressed as the percentage of force generated in response to norepinephrine.

### Histology Staining

Mice abdominal artery were embedded in paraffin after fixed in 4% phosphate-buffered formalin, then achieved 4 μm thick tissue sections. For histological analysis, sections were stained with H&E or Verhoeff–Van Gieson.

### Measurement of Atherosclerotic Lesions

Mice were killed using an overdose of ethyl ether and perfused with PBS. To assess the development of atherosclerosis, the thoracic/abdominal aortas separated from fat or other tissues were stained with Oil Red O (Sangon, Shanghai, China) for 90 min; aortic roots frozen sections by optimal cutting temperature embedding were stained with Oil Red O for 30 min. Imaging software (Image Pro Plus 6.0) was used to measure aortic lesions using the “en face” method, as previously described (Zheng et al., 2016).

### Immunohistochemistry

For immunohistochemistry, dewaxed aortic root sections were fixed with cold acetone for 10 min, then incubated with mouse anti-CD68 (1:100, Abcam) at 4°C overnight. After washing three times with PBS, sections were incubated with goat anti-mouse secondary antibodies (Santa Cruz Biotechnology, United States) at 37°C for at least 1 h. Protein expression was visualized using 3,3′-diaminobenzidine (Vector Laboratories, CA) for 1.5 min, and hematoxylin was used to stain the nuclei.

For immunohistochemistry staining of aortic sections by anti-CD68 and α-smooth muscle actin (α-SMA), dewaxed aortic sections were boiled in 10 mM citrate (pH 6.0) for antigen retrieval, then incubated with mouse anti-CD68 (1:100, Abcam, Ab31630) and mouse anti-α-SMA antibody (1:200, Sigma, A2547) at 4°C overnight. After washing three times with PBS, sections were incubated with goat anti-mouse secondary antibodies (Santa Cruz Biotechnology, United States) at 37°C for at least 1 h. Protein expression was visualized using 3,3′-diaminobenzidine (Vector laboratories, CA) for 1.5 min, and hematoxylin was used to stain the nuclei.

### RNA Extraction and Real-Time Polymerase Chain Reaction

RNA was extracted from mice aorta using an RNA extraction kit (HiScript II 1st strand cDNA Synthesis Kit) purchased from Vazyme Biotech Co., Ltd., according to the manufacturer’s recommended protocol (Weng et al., 2019). A reverse transcription kit (Vazyme, ChamQ SYBR qPCR Master Mix) was used for reverse transcription. Complementary DNA was amplified and measured using a StepOnePlus system (Applied Biosystems). Quantitative qPCR primer sequences were as follows: IL-6, forward 5′-TAGTCCTTCCTACCCCAATTTCC-3′ and reverse 5′-TTGGTCCTTAGCCACTCCTTC-3′; IL-1β forward 5′-TTAA AAACCTGGATCGGAACCAA-3′ and reverse 5′-GCATTAGCT TCAGATTTACGGGT-3′; TNF-α, forward 5′-ATGGGCTGTGA TCGGAACTG-3′ and reverse 5′-GTCTTCCCAATAAGCATGT CTCC-3′. Ccl2 forward 5′-TTAAAAACCTGGATCGGAACC AA-3′ and reverse 5′-GCATTAGCTTCAGATTTACGGGT-3′; Ccl5 forward 5′-GCTGCTTTGCCTACCTCTCC-3′ and reverse 5′-TCGAGTGACAAACACGACTGC-3′; Ccl9 forward 5′-CCCT CTCCTTCCTCATTCTTACA-3′ and reverse 5′-AGTCTTGAA AGCCCATGTGAAA-3′; 18s rRNA forward 5′-CATTCGAACG TCTGCCCTATC-3′ and reverse 5′-CCTGCTGCCTTCCTTG GA-3′. Collagen I forward 5′-GCTCCTCTTAGGGGCCACT-3′ and reverse 5′-CCACGTCTCACCATTGGGG-3′; Collagen III forward 5′-CTGTAACATGGAAACTGGGGAAA-3′ and reverse 5′-CCATAGCTGAACTGAAAACCACC-3′. All PCR primers were purchased from Sangon. Quantitative measurements were obtained using the ΔCt method, using 18s rRNA as an internal control.

### Cell Culture

Human endothelial cell line Ea.hy926 was cultured in Dulbecco’s modified Eagle medium (DMEM) supplemented with 10% fetal bovine serum at 37°C in a 5% carbon dioxide incubator. Small interfering RNAs were purchased from Dharmacon. Transient transfection was performed with Lipofectamine 2000 (Invitrogen, United States, 11668019). Cells were harvested 48 h after transfection. TNF-α was purchased from Peprotech (United States, 300-01A-50). PFI-3 was purchased from Selleck (Shanghai, China, S7315). Ea.hy926 was seeded at 1 × 10^5^ cells/p35 culture dish and starved in serum-free DMEM overnight. TNF-α (10 μg/L) was added the next day for another 12 or 24 h. In certain experiments, PFI-3 (2 μM) was added together with TNF-α.

### Western Blot Technology

For Western blot, the total protein was extracted from Ea.hy926 cells receiving different treatments with radioimmunoprecipitation assay lysis (Beyotime, Shanghai, China) according to the recommended protocol (Li et al., 2020). Thirty micrograms of protein samples were separated on 10% sodium dodecyl sulfate-polyacrylamide gel electrophoresis and transferred to nitrocellulose filter membranes (Merck Millipore, Canada). After being blocked with 5% non-fat milk for 1 h at room temperature, the membranes were incubated with anti-TBP antibody (1:2,000 dilution, YIFEIXUE BIO TECH, A0055), anti-Poly II antibody (1:2,000 dilution, Proteintech, 20655-I-AP), anti-BRG1 antibody (1:2,000 dilution, Abcam, Ab110641), and anti-c-Jun and anti-c-Fos antibody (1:500 dilution, Santa Cruz, sc-1694 or sc-52) overnight at 4°C. The Anti-Poly II antibody or anti-TBP antibody was used as a standard internal protein for normalizing. Horseradish peroxidase-conjugated immunoglobulin G (YIFEIXUE BIO TECH, China) was used to amplify the signal. After treatment with the chemiluminescence kit (Thermo Fisher Scientific, United States), protein signals were detected by ChemiDoc RXC + (Bio-Rad, United States).

### Immunofluorescence Staining

Briefly, cells were plated at a density of 2 × 10^4^ cells per dish. After treatment with TNF-α, the cells were washed with PBS three times, fixed by 1% paraformaldehyde for 10 min, and stained with a c-Fos antibody (Sigma, United States A7811, 1:200) overnight at 4°C. The next day, the cells were incubated with AF488-labeled secondary antibody (Jackson ImmunoResearch) for 1 h. The nuclei were counterstained with 4’,6-diamidino-2-phenylindole (Sigma, United States). Immunofluorescence was visualized on a confocal microscope (LSM 710, Zeiss).

### Electrophoretic Mobility Shift Assay

Nuclear proteins for electrophoretic mobility shift assay (EMSA) were achieved according to the manufacturer’s instruction. The nuclear proteins (5 μg) of each group were incubated with 1 × binding buffer (LightShift Chemiluminescent EMSA Kit, Pierce) in the presence of 50 ng/μl poly (dI/dC), 0.05% non-idet P-40, 5 mM MgCl_2_, and 2.5% glycerol for 10 min and then incubated at room temperature for additional 20 min with 1 pmol of biotin-labeled Ap-1 oligonucleotide (Sangon Biotech Co., Ltd.). The reaction mixture was subjected to a 6% non-denaturing sodium dodecyl sulfate-polyacrylamide gel electrophoresis at 100 v for 50 min, transferred to polarity nylon hybridization transfer membrane (Beyotime, China) and DNA cross-linked for 2 min, and probed with peroxidase-conjugated streptavidin antibodies (1:1,500 dilution, Bytotime, China), then visualized with enhanced chemiluminescence and detected by ChemiDoc RXC + (Bio-Rad, United States). The sequences of Ap1 probe and mutant probe are listed: probe forward 5′-ATTTGTTCGGGGCGGGGCGAGC-3′; probe reverse 3′-TAAACAAGCCCCGCCCCGCTCG-5′.

### Luciferase Report Assay

293T cells were transfected using Lipofectamine 2000 (Invitrogen, United States) in serum-free DMEM media with MCP-1-Luc plasmid (0.2 μg) and transfected with BRG1 overexpression adenoviruses. Six hours after transfection, the cells were culture in DMEM containing 10% fetal bovine serum for 24 h; the cells were lysed, and luciferase activity was determined by using the luciferase assay system according to the manufacturer’s instruction (Promega Corp., United States). The MCP-1-Luc plasmid promoter fragments span the 2,000(−2,000) bp by region upstream of the transcription site. For controlling for differences in transfection efficiency, a plasmid that contained green fluorescent protein fluorescence was included in each transfection and used for normalization.

### Statistical Analysis

Significant differences between the two groups were analyzed by unpaired Student’s *t*-test (GraphPad Prism software, version 5.0; GraphPad Prism, United States). All experiments were performed with similar results, at least in triplicate repeat. Data are expressed as the mean ± SD. Statistical significance was set to *P* < 0.05.

## Results

### Specific Deletion of Brahma-Related Gene 1 in Endothelial Cells Alleviated Diabetes Mellitus-Related Atherosclerosis

**FIGURE 1:**
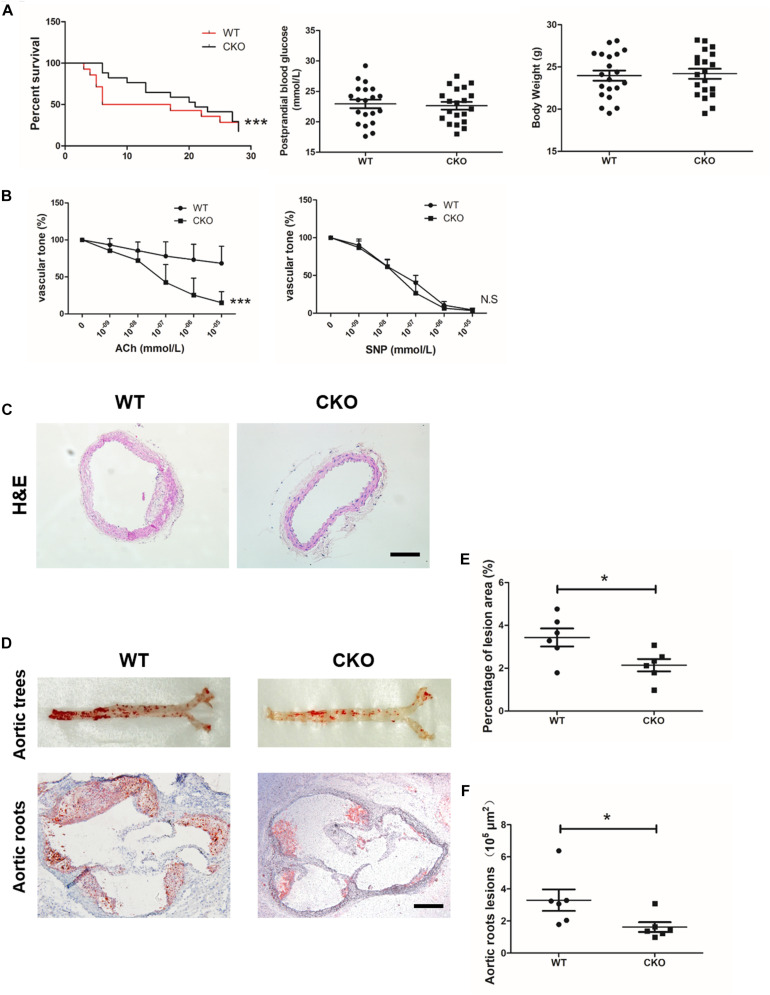
Endothelial cell-specific deletion of BRG1 alleviated diabetes mellitus-related atherosclerosis. **(A)** Survival rate, body weight, and postprandial blood glucose for STZ and Western diet-induced mice diabetic atherosclerosis (*n* = 20). **(B)** Vascular tone experiment to detect the vasodilation effect of aortic after acetylcholine or SNP treatment. **(C)** H&E staining for abdominal aortic. **(D)** Oil-red O staining for aortic trees and aortic roots. **(E,F)** Statistics for Oil-Red O staining (scale bar: 200 μm, *P < 0.05 and ***P < 0.001).

Diabetic atherosclerosis was induced in ApoE*^–/–^*; BRG1*^*fl/fl*^*; CDH5*-cre* mice (CKO) and their littermate control ApoE*^–/–^*; BRG1*^*fl/fl*^* (WT) mice to evaluate the effects of endothelial-specific BRG1 deletion *in vivo*. As shown in Figure 1A, there was a significant mortality rate reduction in the ApoE*^–/–^*; BRG1*^*fl/fl*^*; CDH5-*cre* group compared with their littermate control group (*p* < 0.001), despite body weight and postprandial blood glucose did not obviously hang between the two groups. *In vitro* vascular tone experiment showed that endothelial BRG1 deletion ameliorated the vasodilation defect after acetylcholine treatment (Figure 1B, *P* < 0.001); vascular tone for SNP treatment were used as the positive control, and there was no difference of aortic relaxation in response to the nitric oxide donor SNP in the two groups. H&E staining of the aortic arteries showed that endothelial BRG1 deletion mitigated the atherosclerotic injuries in mice (Figure 1C). Meanwhile, Oil Red O staining, which allows the visualization of lipids, indicated over 30% reduction of atherosclerotic lesions (*p* = 0.0263) in the aortic trees (Figures 1D,E) and about 50% reduction (*p* = 0.0453) in the aortic roots (Figures 1D,F) in CKO mice compared to WT mice. These combined results suggest that endothelial-specific BRG1 deficiency may counteract the progression of diabetic atherosclerosis in mice.

### Brahma-Related Gene 1 Deletion in Endothelial Cells Reduced Plaque Inflammatory Levels and Oxidative Stress in Diabetic Mice

**FIGURE 2:**
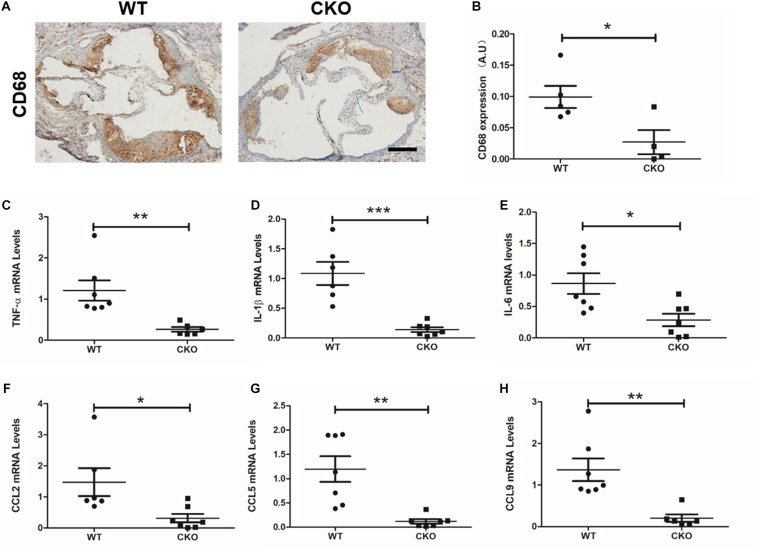
BRG1 deletion in endothelial cells reduced plaque inflammatory levels and oxidative stress in diabetic mice. **(A)** CD68 immunohistochemistry staining for aortic roots and **(B)** statistics. **(C)** TNF-α, **(D)** IL-1β and **(E)** IL-6 mRNA levels detected by real-time PCR in abdominal/thoracic aortic. **(F–H)** Real-time PCR results for CCL2, CCL5, and CCL9 in abdominal/thoracic aortic (Scale bar: 200 μm, *P < 0.05, **P < 0.01, and ***P < 0.001).

Macrophage recruitment is one of the key characteristics during the development of atherosclerosis. As shown in Figures 2A,B, weaker CD68 immunohistochemistry staining was observed in the CKO plaques than in the WT plaques. Meanwhile, real-time quantitative PCR assay demonstrated that expression levels of a panel of pro-inflammatory mediators, including TNF-α (Figure 2C), IL-1β (Figure 2D), IL-6 (Figure 2E), CCL2 (Figure 2F), CCL5 (Figure 2G), and CCL9 (Figure 2H), were downregulated in the CKO mice compared with the WT mice. These data suggest that endothelial BRG1 may contribute to diabetic atherosclerosis by modulating vascular inflammation.

### Endothelial Deletion of Brahma-Related Gene 1 Ameliorates Ang II-Induced Abdominal Aortic Aneurysm Progression in Mice

**FIGURE 3:**
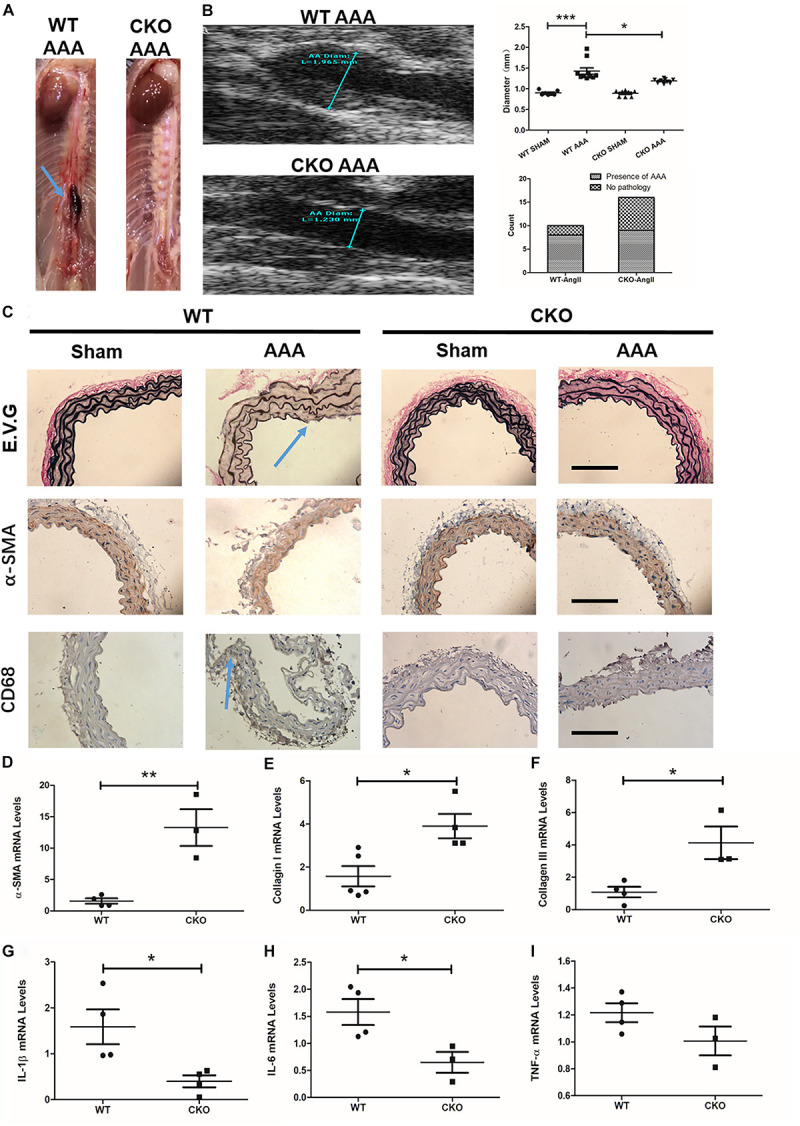
Endothelial cell deletion of BRG1 ameliorates angiotensin II-induced AAA progression in mice. **(A)** Gross anatomy of abdominal aortas at 4 weeks. **(B)** Echocardiographic analysis of vessel diameters at 4 weeks. **(C)** Verhoeff–Van Gieson elastic staining, α-SMA staining, and CD68 staining in AAA, **(D)** α-SMA, **(E)** collagen-I, and **(F)** collagen-III mRNA levels in abdominal aortic. **(G)** IL-1β mRNA levels detected by real-time PCR in abdominal/thoracic aortic. **(H)** IL-6 mRNA levels detected by real-time PCR in abdominal/thoracic aortic. **(I)** TNF-α mRNA levels detected by real-time PCR in abdominal/thoracic aortic (Scale bar: 200 μm, *P < 0.05, **P < 0.01, and ***P < 0.001).

We next determined the role of endothelial BRG1 in Ang II-induced AAA model in CKO mice and WT mice. Of interest, 4 weeks after the Ang-II infusion, the gross anatomical evaluation revealed that the abdominal aortas in WT mice became overtly enlarged, indicative of AAA development (Figure 3A); by comparison, mice with endothelial conditional Brg1 knockout (CKO) exhibited an appreciable reduction in aortic enlargement. The ultrasonographic examination confirmed that Brg1 deficiency in endothelial cells led to an approximately 10% reduction in aortic diameter (Figure 3B). The incidence of AAA found on autopsy was 8/10 in the Ang II infusion WT group compared with 9/16 in the Ang II infusion KO group (Figure 3B). Morphometric analyses by Verhoeff–Van Gieson elastic staining showed significant dilation or breakage of the external and internal aortic walls in ApoE*^–/–^;* BRG1*^*fl/fl*^* mice induced by Ang II (Figure 3C). α-SMA immunohistochemistry staining indicated an alleviation of elastin fragmentation in ApoE*^–/–^*; BRG1*^*fl/fl*^*; CDH5*-cre* mice treated by Ang II (Figure 3C). Meanwhile, CD68 immunohistochemistry staining also cues less macrophage recruitment in Ang II-infused CKO mice (Figure 3C). Further detection proved the significant upregulation of α-SMA (Figure 3D), collagen I (Figure 3E), and collagen III (Figure 3F) in the ApoE*^–/–^*; BRG1*^*fl/fl*^*; CDH5*-cre* mice, suggesting that endothelial cell deletion of BRG1 could ameliorate Ang II induced AAA progression in mice.

Pro-inflammatory factors participate in many important processes of AAA progression. To further determine the role of endothelial BRG1 in an aneurysm, pro-inflammatory cytokines, such as TNF-α, IL-1β, and IL-6, were measured by real-time qPCR. The data shown in Figure 3 indicated that IL-1β and IL-6 messenger RNA (mRNA) levels significantly decreased in the ApoE*^–/–^*; BRG1*^*fl/fl*^*; CDH5*-cre* mice group (Figures 3G,H). However, there seemed to be no significant alteration of TNF-α (Figure 3I) between the two groups, indicating that endothelial BRG1 deletion could alleviate AAA progression via regulating IL-1β and IL-6 levels.

### Inhibition of Brahma-Related Gene 1 Reduced Inflammatory Response and c-Fos Expression in Endothelial Cells

**FIGURE 4:**
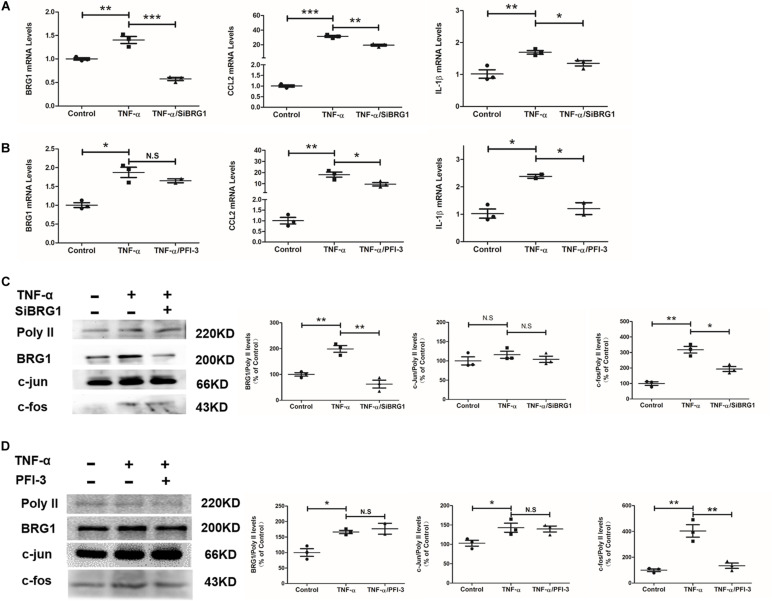
Inhibition of BRG1 reduced inflammatory response and c-Fos expression in endothelial cells. **(A)** Ea.hy926 cells were transfected with siRNA targeting BRG1 or scrambled siRNA (SCR) followed by treatment with TNF-α (10 μg/L) for 24 h. Gene expression was examined by qPCR. **(B)** Ea.hy926 cells were transfected with PFI-3, followed by treatment with TNF-α (10 μg/L) for 24 h. Gene expression was examined by qPCR. **(C)** Ea.hy926 cells were transfected with siRNA targeting BRG1 or scrambled siRNA (SCR), followed by treatment with TNF-α (10 μg/L) for 24 h. Gene expression was examined by Western. **(D)** Ea.hy926 cells were transfected with PFI-3, followed by treatment with TNF-α (10 μg/L) for 24 h. Gene expression was examined by Western (*P < 0.05, **P < 0.01, and ***P < 0.001).

Regarding an inflammatory factor, TNF-α would increase inflammatory response in endothelial cells *in vitro*. In this work, exposure of Ea.hy926 cells to TNF-α increases the mild but obvious BRG1 upregulation. Interestingly, BRG1 inhibition significantly reduced BRG1 expression, which downregulates together with IL-1β and CCL2 in Ea.hy926 cells after anti-SiBRG1 RNAi treatment (Figure 4A). Despite not altering BRG1 mRNA levels, a molecular inhibitor of BRG1, PFI-3 treatment significantly decreased IL-1β and CCL2 expression after TNF-α treatment (Figure 4B). Transcription factor AP-1 is a menagerie of dimeric basic region leucine zipper proteins, consisting of homodimers of Jun or heterodimers of c-Fos and c-Jun. To explore the potential involvement of AP-1 in decreasing inflammatory factors by BRG1 inhibition, Figures 4C,D show significantly decreased c-Fos protein levels by anti-SiBRG1 RNAi or PFI-3 treatment in TNF-α-inducing endothelial cells *in vitro*. These data strongly suggest that blocked BRG1 functions obviously alleviated TNF-α-induced inflammatory response.

### Inhibition of Brahma-Related Gene 1 Blocked c-Fos Translocation in Endothelial Cells

**FIGURE 5:**
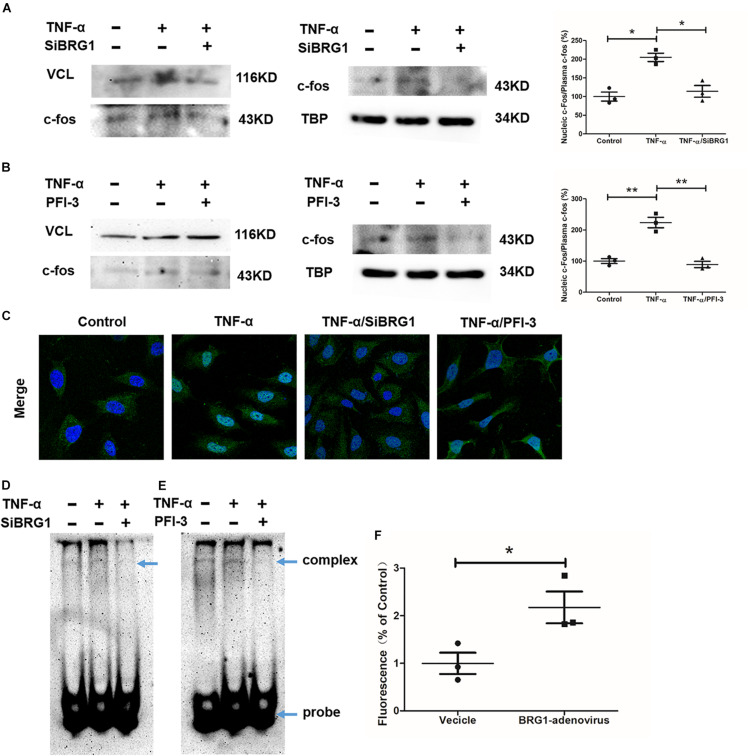
BRG1 inhibition blocked c-Fos translocation in endothelial cells. **(A)** Ea.hy926 cells were transfected with siRNA targeting BRG1 or scrambled siRNA (SCR), followed by treatment with TNF-α (10 μg/L) for 24 h. C-Fos translocation levels were examined by Western. **(B)** Ea.hy926 cells were transfected with PFI-3, followed by treatment with TNF-α (10 μg/L) for 24 h. C-Fos translocation levels were examined by Western. **(C)** Immunofluorescence staining was performed with anti-c-Fos antibody. **(D)** Ea.hy926 cells were transfected with siRNA targeting BRG1 or scrambled siRNA (SCR), followed by treatment with TNF-α (10 μg/L) for 24 h. Alternatively, **(E)** Ea.hy926 cells were treated with TNF-α (10 μg/L) and PFI-3 for 24 h. Nuclear lysates were extracted, and EMSA was performed as described in section “Materials and Methods.” **(F)** 293T cells were co-transfected with plasmid containing MCP-1-promoter-luc and overexpression Sp1 adenoviruses. Luciferase report assay was performed as described in section “Materials and Methods.” (*P < 0.05 and **P < 0.01).

We measured the effect of c-Fos translocation into nuclear by Western blot, as shown in Figures 5A,B. c-Fos translocation into nuclear was significantly reduced by TNF-α induction after anti-SiBRG1 RNAi or PFI-3 treatment. In accordance, immunofluorescent staining of c-Fos showed that both siRNA-mediated depletion of BRG1 and PFI-3-mediated inhibition of BRG1 (Figure 5C) reduced TNF-α-induced c-Fos nucleic translocation of EA.Hy926 cells. AP-1 activation is also involved in increased DNA binding activity. We performed EMSA using a biotin-labeled double-strand robe corresponding to AP-1 and its flanking sequence. The DNA-AP-1 complex formation was induced obviously by TNF-α treatment, and the complex induced by TNF-α was significantly reduced when the cells were treated with anti-SiBRG1 RNAi or PFI-3 (Figures 5D,E). Luciferase report assay results in Figure 5F proved that the effect of MCP-1 promoter activation was induced by BRG1 overexpression in 293T cells. In Figure 5F, the induction of luciferase activity of MCP-1 also increased after BRG1 overexpression adenovirus transfection.

## Discussion

BRG1, encoded by the *SMARCA4* gene, is a member of the SWI/SNF family and shares significant homology with the Drosophila Brahma protein. BRG1 possesses an intrinsic ATPase activity and participates in regulating gene transcription by altering the chromatin structure. Recent research indicates that genetic interference of BRG1 expression in the adult mouse may bring beneficial effects in the models of CVD. For instance, it has been reported that BRG1 interference protected the mice against cardiac ischemia–reperfusion (Li Z. et al., 2018; Zhang et al., 2018b) and atherosclerosis (Fang et al., 2013). In contrast, endothelial cell-specific deletion of BRG1, achieved by lentivirus-mediated delivery of short hairpin RNA, resulted in anti-inflammatory effects via inhibiting inflammatory factors such as TNF-α in mice, which further ameliorated atherosclerosis in mice models (Fang et al., 2013). In the present study, we aimed to explore the role of endothelial BRG1 in diabetic atherosclerosis and AAA, and the results demonstrated the deletion of BRG1 in endothelial cells, which contributed to improved survival rate and amelioration of vascular function in a model of diabetic atherosclerosis. In addition, endothelial cell conditional BRG1 deletion also blocked the progression of AAA in mice.

Among the diabetic complications, vascular damage contributes to the major burden of morbidity and mortality associated with diabetes. In this research, we reported the deletion of endothelial BRG1 that ameliorated the survival rate in a model of diabetic-related atherosclerosis. Hyperglycemia is a risk factor for atherosclerotic disease. In this model, STZ has been utilized to induce mice hyperglycemia. According to previous studies, mice containing atherogenic mutations in ApoE^–/–^ mice develop either spontaneous or high fat diet-induced coronary artery atherosclerosis, myocardial infarction, and dramatically reduced survival. This reduced survival of these mice seems to be associated with myocardial infarction and resulting cardiac conduction and functional abnormalities (Nakatsu et al., 2017; Wang Q. et al., 2017; Gonzalez et al., 2018). However, these associations still need to be further investigated. In many instances, the underlying pathology of diabetic atherosclerosis is observed in diabetic patients with myocardial infarction or thromboembolic stroke (Tillquist and Maddox, 2012). Along with heart failure and coronary artery disease, endothelial dysfunction has been considered as a key etiological factor for the progression of diabetic-induced atherosclerosis (Kovacic et al., 2014). Recent studies have suggested that attenuation of inflammation-related endothelial dysfunction could be considered as a reasonable therapeutic approach for diabetic atherosclerosis treatment. For instance, it is well known that inhibiting inflammatory factors such as IL-1β and IL-6 secreted by injured endothelium could protect atherosclerotic lesions (Alfaidi et al., 2018). Interestingly, dampened expression of C-C motif chemokine ligands helps diabetes-associated atherosclerosis (Cao et al., 2014; Xuan et al., 2017; Lin et al., 2018). BRG1 has been reported to participate in the regulation of inflammatory progression in many diseases progression. For instance, hepatocyte-specific Brg1 deletion alleviated the progression of steatohepatitis via regulating SREBP activity-related inflammatory response (Li N. et al., 2018). In cardiac ischemia–reperfusion injury mouse models, endothelial conditional Brg1 deficiency plays a protective role in ameliorating heart function through blocked neutrophil recruitment (Zhang et al., 2018b). This research indicated the promising effect of anti-inflammatory treatment by BRG1 deletion. The present study explores the anti-inflammatory effect of endothelial conditional BRG1 deletion in a diabetic atherosclerosis model in mice. We found that BRG1 deletion in endothelial cells reduced atherosclerotic plaques in thoracic/abdominal aortic and aortic roots in mice. Meanwhile, the deletion of BRG1 in endothelial cells reduced macrophage recruitment in plaques. Further research proved a reduction in IL-1β, IL-6, CCL2, CCL5, and CCL9 mRNA levels in BRG1 CKO mice. These findings provide novel insights that BRG1 is linked to diabetic atherosclerosis via a regulated inflammatory response in endothelial cells.

AAA is one of the most deadly cardiovascular pathologies. Although it is typically regarded as being a distinct entity, vascular inflammation also acts as a common pathogenic factor for AAA. Recent research indicated that a dysfunctional endothelium could increase ROS levels or secretion of chemokines and cytokines, which finally influence the progression of AAA (Siasos et al., 2015). Ameliorating endothelium function has been considered as a promising target for AAA treatment. The current report indicated that statins still seem very promising in animal AAA models via improving endothelial function (de Sotomayor et al., 2005). In addition to their lipid-lowering capacity, statin can improve endothelial function via increasing endothelial nitric oxide synthase expression and BH4 bioavailability (Kosmidou et al., 2007; Wenzel et al., 2008). In contrast, statin treatment can also diminish IL-6 and MCP1 levels in AAA models (Kowalska et al., 2018). In this study, we focused on the protective effects of endothelium-specific BRG1 deletion in AAA, and our results demonstrated that BRG1 deletion in endothelial cells significantly inhibited Ang II-induced AAA development. Deletion of BRG1 in endothelial cells also augmented collagen I, collagen III, and α-SMA levels in mice. These findings collectively argue that BRG1 in endothelial cells could promote AAA pathological progression. Further studies showed a significant reduction of IL-1β and IL-6 but not TNF-α expression in mice, which indicated BRG1 might promote AAA by stimulating endothelial-derived pro-inflammatory mediators. Alternatively, the changes in expression levels of pro-inflammatory mediators could be secondary to a reduction in the recruitment of immune cells (e.g., macrophages).

Previous research reported endothelial-specific BRG1 knockout attenuated the progression of CVDs. For example, Weng et al. (2015) demonstrated endothelial-specific knockdown BRG1 ameliorating cardiac hypertrophy both *in vitro* and *in vivo*. Meanwhile, endothelial-specific BRG1 knockout had also been proved to ameliorate the CaCl_2_-induced mice AAA progression via trans-activates endothelium-derived colony-stimulating factor (Zhang et al., 2018a). However, the mechanism of BRG1 in regulating Ang-II-induced AAA formation under *ApoE* gene knockout condition remains not to be proven. In this research, we reported endothelial-specific BRG1 knockout reduced AAA progression in mice Ang-II-induced AAA models.

We further reported that BRG1 regulated c-Fos expression and nucleic translocation in endothelial cells. In EA.hy926 cells, it obviously decreased c-Fos expression and blocked c-Fos translocation after anti-SiBRG1 RNAi or PFI-3 treatment. Protein c-Fos belongs to transcription factor AP-1, a menagerie of dimeric basic region−leucine zipper proteins, consisting of homodimers of Jun or heterodimers of Fos and Jun (Shaulian and Karin, 2001, 2002). Recent research reported the effects of regulating inflammatory-related gene expression by AP-1. A binding site of AP-1 in IL-1β and CCL2 promoter region has been identified in a variety of cell types, and AP-1 is an important transcription factor regulating inflammatory-related gene expression (Morse et al., 2003; Sutcliffe et al., 2009). AP-1 is a mediator of inflammatory responses and activated by TNF-α in endothelial cells, and TNF-α-induced endothelial inflammatory response is dependent on AP-1 activation (Morse et al., 2003; Hong et al., 2016). Together, these pieces of evidence suggest that activation of AP-1 enhances inflammatory response in endothelial cells under TNF-α treatment conditions. In this research, we further demonstrated endothelial conditional BRG1 knockout could not alter the c-Jun expression. Among these studies, we inched that the mechanism of BRG1 regulated inflammatory factors expression via mediated by c-Fos nucleic translocation, which could finally block the progression of diabetes-related atherosclerosis and Ang-II-induced mice AAA progression.

Adding to the understanding of the endothelial protective activity of PFI-3 in endothelial cells, we presented pieces of evidence that blocked the function of BRG1-inhibited IL-1β and CCL2 expression and decreasing BRG1 function also inhibiting the activity of AP-1 in endothelial cells. We showed that treatment with PFI-3 blocked the regulation of IL-1β and CCL2 by TNF-α through inhibition of the IL-1β and CCL2 gene promoter. Accompanying the suppression of c−Fos translocation, decreasing the BRG1 function also inhibited the DNA binding activity of AP-1 demonstrated by EMSA assays. Because the IL-1β and CCL2 promoter contains an AP-1−binding site (Morse et al., 2003; Sutcliffe et al., 2009), it is highly likely that the effect of PFI-3 on blocking TNF-α, which lead to induced IL-1β and CCL2 expression, is through the regulation of c-Fos promoter activity.

In summary, our results reveal a previously unrecognized protective role of endothelial cell-specific BRG1 deletion in alleviating diabetic atherosclerosis and Ang II-induced AAA progression via inhibiting the inflammatory responses. These findings indicated that BRG1 could be targeted to alleviate endothelial dysfunction, which helps diabetic atherosclerosis and AAA treatment.

## Data Availability Statement

The raw data supporting the conclusions of this article will be made available by the authors, without undue reservation.

## Ethics Statement

The studies involving animals were reviewed and approved by the intramural Committee on Ethical Conduct of Animal Studies of Nanjing Medical University.

## Author Contributions

TL and TW conceived the project. YZ and TW designed the experiments. TW, YZ, TX, and XC performed the experiments and collected data. TW wrote the manuscript. YX and TL secured funding and provided supervision. HW, MS, and TW added the experiments in revised manuscript.

## Conflict of Interest

The authors declare that the research was conducted in the absence of any commercial or financial relationships that could be construed as a potential conflict of interest.
